# Study of the Optical, Structural and Electrophoretic Properties (Zeta Potential and Hydrodynamic Diameter) of SiO_2_-Coated Ag Nanoparticles

**DOI:** 10.3390/nano16030212

**Published:** 2026-02-06

**Authors:** Víctor E. Gámez-Albo, Ana B. López-Oyama, Eugenio Rodríguez González, Jesús R. González-Castillo, Daniel Jímenez-Olarte, Deyanira Del Ángel-López, Elizabeth Reyna-Beltrán, Edgar G. Zamorano-Noriega

**Affiliations:** 1 Centro de Investigación en Ciencia Aplicada y Tecnología Avanzada-Unidad Altamira, Instituto Politécnico Nacional, Km 14.5 Carr. Puerto Industrial, Altamira 89600, Tamaulipas, Mexico; vgameza1900@alumno.ipn.mx (V.E.G.-A.); ddelangel@ipn.mx (D.D.Á.-L.); 2 Departamento de Investigación en Física (DIFUS), Universidad de Sonora, Blvd. Transversal S/N, Hermosillo 83000, Sonora, Mexico; 3 Secihti-DIFUS, Universidad de Sonora, Blvd. Transversal S/N, Hermosillo 83000, Sonora, Mexico; 4 Escuela Superior de Física y Matemáticas (ESFM—IPN), Instituto Politécnico Nacional, Mexico City 07738, Mexico; jrgonzalezc@ipn.mx; 5 Escuela Superior de Ingeniería Mecánica y Eléctrica (ESIME—IPN), Instituto Politécnico Nacional, Mexico City 07738, Mexico; dajimenez@ipn.mx; 6 Facultad de Medicina “Dr. Alberto Romo Caballero”, Universidad Autónoma de Tamaulipas, Tampico 87000, Tamaulipas, Mexico; ereyna@docentes.uat.edu.mx; 7 Departamento de Investigación en Polímeros y Materiales (DIPM), Universidad de Sonora, Blvd. Transversal S/N, Hermosillo 83000, Sonora, Mexico; a207217671@unison.mx

**Keywords:** Ag@SiO_2_ nanoparticles, laser ablation, REDOX reactions, zeta potential, colloidal stability

## Abstract

Colloidal solutions containing silica-coated silver nanoparticles (Ag@SiO_2_) were synthesized through a two-step process integrating physical and chemical mechanisms. In the first step, laser ablation of a silicon target submerged in deionized water generated an H_2_O–SiO_2_ colloid, termed the as-cast colloid. This contained nanometric SiO_2_ particles alongside micrometer-sized or larger silicon fragments produced by laser shockwave-induced target surface fragmentation. To refine particle size distribution and elevate nanometric SiO_2_ concentration, the as-cast colloid underwent secondary laser irradiation, effectively fragmenting larger particles. The second step involved adding ionic silver to both as-cast and irradiated colloids, yielding Ag@SiO_2_ nanoparticles. Structural properties were probed via XRD and TEM; optical characteristics via UV–Vis spectroscopy; and electrophoretic mobility via zeta potential measurements, both pre- and post-silver incorporation, to elucidate irradiation’s influence on synthesis. For controlled agglomeration, AlCl_3_ was used to modify surface charge, neutralizing silanol groups on the silica shell and minimizing electrostatic repulsion through aluminum ion interactions. These findings demonstrate tunable Ag@SiO_2_ colloids with precise surface properties for future development of advanced nanomaterials suitable for microbicidal applications.

## 1. Introduction

In the past few years, metal nanoparticles have garnered the attention of researchers due to their extraordinary potential applications. The production of metallic nanoparticles has been achieved through a variety of chemical methods, including physical–chemical reduction [1,2] chemical reduction with both organic [3,4] and inorganic reagents [5,6], condensation, and evaporation [7,8].

A variety of metal nanoparticles with distinct antimicrobial mechanisms have been examined for their microbicidal properties, including silver nanoparticles (AgNPs) [9]. Numerous studies have demonstrated their efficacy against a variety of pathogens [10,11,12]. A key feature of AgNP’s is the surface plasmon resonance (SPR), a phenomenon caused by the coherent oscillation of the free electrons in response to an external electromagnetic field such as light. This resonance generates a strong electric field surrounding the nanoparticle which can result in a variety of effects, such as the generation of reactive oxygen species (ROS), the ejection of “bullet-like” atoms or clusters, and an increase in light absorption, all of which contribute to their microbicidal activity [13,14,15]. Despite their promising properties, AgNP commonly face challenges related to instability and reactivity in aqueous media, limiting their practical applications. To overcome these issues, AgNPs are often coated with stabilizing materials that reduce core reactivity without compromising their optical properties. In this regard, silicon dioxide (SiO_2_) has emerged as a noble coating material due to its chemical stability, transparency, and easy of synthesis [16].

Recently, laser ablation has garnered substantial attention in addition to the chemical routes for nanoparticles fabrication [17,18,19]. This technique enables the fabrication of a large variety of nanoparticles both in colloids [20], and under vacuum conditions [21,22,23].

In this context, a two-step process integrating laser ablation and redox reactions has been reported for the synthesis of silica-coated silver nanoparticles (Ag@SiO_2_ NPs) [24]. The initial step involves laser ablation of a silicon target submerged in water. During this stage, silicon nanoparticles are generated and rapidly oxidize, resulting in the formation of an H_2_O–SiO_2_ nanoparticle colloid. In the subsequent step, silver ions are introduced into the colloid, resulting in the formation of silver nanoparticles that are coated by a silica shell, and thus producing Ag@SiO_2_ NPs [25,26]. The optical, structural, and photo-induced microbicidal properties of these Ag@ SiO_2_ nanoparticles have been reported to be effective against both human bacteria [27] and phytopathogens [28]. However, during the first step—laser ablation of the Si target—in addition to nanoscale SiO_2_ particles, larger micro- and submicron-sized silicon particles are also generated because of laser shockwave-induced fragmentation of the target surface. These larger particles do not fully oxidize in water and precipitate within minutes, thereby compromising colloidal stability, coating uniformity, and ultimately reducing the efficiency of the fabrication process.

To address this issue, the present study introduces a previously unreported modification consisting of an additional laser irradiation step applied to the as-prepared (as-cast) H_2_O–SiO_2_ colloid. This treatment aims to fragment larger silicon particles, thereby improving colloidal homogeneity and stability, while simultaneously modifying the structural and electrophoretic properties of the system. To the best of our knowledge, this synergistic effect has not been reported in the literature.

A key factor in enhancing microbicidal efficacy is the precise regulation of the electrostatic interactions between microorganisms and nanoparticles. Subsequently, to facilitate electrostatic interaction, nanoparticles must include neutral or positive surface charges [29]. The antimicrobial activity of silver nanoparticles coated with silicon dioxide is substantial against bacterial strains, including both Gram-positive and Gram-negative species. Electrostatic interactions between the positively charged or neutrally stabilized Ag@SiO_2_ nanoparticles and the negatively charged bacterial cell wall promote adhesion to the microbial surface. The silica coating ensures a high surface area, available for interaction by enhancing nanoparticle stability and preventing agglomeration [30]. The microbial efficacy is primarily attributed to both the plasmonic effect and the release of Ag^+^ ions from the silver core, which could bind to sulfur and phosphorous-containing biomolecules within the bacterial membrane and intracellular environment. This behavior disrupts membrane integrity and inhibits critical metabolic processes. So far, the nanoparticles’ high surface area enables them to establish a close relationship with the bacterial membrane, thereby enhancing these effects. Silver exposure can be optimized to enhance antimicrobial performance through optimizations of the silica shell and taking advantage of the surface plasmon resonance. Ag@SiO_2_ nanoparticles are promising candidates for applications in antimicrobial coatings and therapies due to their exceptional bactericidal mechanisms and physicochemical stability.

Moreover, certain microorganisms, such as fungi, can attain sizes of tens of microns, necessitating the controlled agglomeration of nanoparticles to facilitate effective treatment coverage. The surface charge can be modulated by the pH and adding salts in varying concentrations (8 × 10^−5^–10^−1^ M) [31,32].

Even though the silica layer is the source of the primary stabilization effect, the optical and electrophoretic properties of Ag@SiO_2_ nanoparticles were examined in a solution of AlCl_3_ salt since AlCl_3_ serves as an agent which promotes agglomeration of nanoparticles [33,34].

Finally, to better understand the fabrication process of the AgSiO_2_ NPs, this research aims to systematically study the structural, optical, and electrophoretic properties of H_2_O–SiO_2_ colloids obtained at different stages of the synthesis, namely, before and after laser irradiation, as well as before and after the addition of the silver salt used to form the AgSiO_2_ nanoparticles.

## 2. Materials and Methods

The laser ablation and irradiation were carried out using a pulsed laser (Nd: YAG, Q-Smart 450Quantel (LUMIBIRD Group), Lannion, France) operating in Q-Switch mode. The laser is equipped with a harmonic generator owning automatic phase correction and operates at a frequency of 20 Hz, delivering 5 ns pulses, with maximum pulse energies of 450 mJ, 200 mJ, and 100 mJ for the first (λ = 1064 nm), second (λ = 532 nm), and third (λ = 355 nm) harmonics, respectively. Laser ablation of the silicon target was performed using the first harmonic of the laser (1064 nm), while the subsequent laser irradiation step was carried out using the second harmonic (532 nm).

Optical properties of samples were studied through UV–Vis spectroscopy within a range of 200 to 800 nm using a UV-VIS Cary 5000 Agilent spectrophotometer (Santa Clara, CA, USA).

The structural properties of SiO_2_-H_2_O colloids and colloids containing Ag@SiO_2_ NPs were studied by X-ray diffraction (XRD) using a Brucker Advance D8 diffractometer (Karlsruhe País: Alemania). The colloidal solution was drop-cast onto a glass substrate heated to 50 °C until enough material was obtained for X-ray detection. The dried powder material was then transferred onto a zero-background silicon sample holder. XRD measurements were performed in the Bragg–Brentano configuration over the 2θ range 20° < 2θ < 80°. The step size was selected to ensure adequate resolution of the diffraction peaks. To enable a proper comparison among the different diffractograms, both the amount of sample and the acquisition time per point were kept constant in all measurements.

TEM and HRTEM micrographs were acquired with a JEOL JEM2100 microscope (Ltd., Tokyo, Japan) at 200 kV. Zeta potential and hydrodynamic diameter were measured using a Litesizer 500 particle analyzer from Anton Paar, Graz, Austria. The pH measurements used to determine the isoelectric point were made with a high-accuracy pH hand tester (HI98130) from Hanna Instruments (Smithfield, Estados Unidos). Colloidal SiO_2_ was measured to evaluate the influence of the irradiation process and colloids containing Ag@SiO_2_ NPs were measured before and after adding different AlCl_3_ concentrations to determine its influence on the nanoparticle’s surface charge and agglomeration.

### 2.1. Experimental System

**Figure 1:**
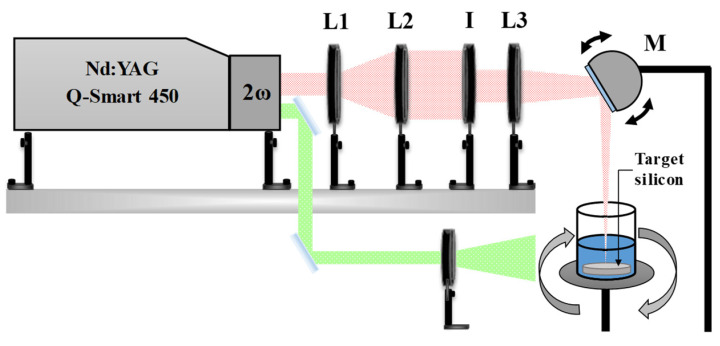
Schematic representation of the laser system used for ablation (red) and irradiation (green).

A schematic representation of the experimental setup used for Ag@SiO_2_ NPs synthesis is illustrated in Figure 1. The laser beam is focused onto the target surface using a converging lens (L3, f3 = +30 cm) and a motorized reflective mirror (M). The mirror oscillates at a fixed frequency (~2 Hz), enabling the focused beam to scan along the central axis of the target. The silicon target is positioned at the bottom of a beaker containing 20 mL of solvent, in this case, HPLC water.

The beaker is rotated around its central axis at ≈60 rpm. This combination of both movements (beam sweeping and container rotation) enables nearly uniform ablation of the target’s surface, thereby mitigating the crater formation resulting from multiple pulses impacting the same point on the target’s surface.

The spot size at the lens focal point was measured using an optical microscope, yielding a diameter of 1 mm. Consequently, the energy density (fluence) at the target surface was calculated based on the pulse energy utilized, resulting in a value of 3.8 J/cm^2^ for a pulse energy (E_P_) of 30 mJ.

The pulse energy was regulated using a metallic iris (I). The laser beam was previously expanded (3×) using a lens arrangement (L1, f1 = −10 cm; L2, f2 = +30 cm) to prevent damage to the iris surface from the laser radiation. Finally, the energy that reaches the target’s surface was calibrated by adjusting the iris aperture. Before every synthesis process, the energy incident on the material surface was measured with a power meter (Ophir Nova II; Ophir Optronics Solutions Ltd., Jerusalén, Israel) equipped with a pyroelectric sensor (PE25BF-DIF-C, Jerusalén, Israel).

### 2.2. Physical Processes: Laser Ablation and Laser Irradiation

SiO_2_ nanoparticles were produced by laser ablation of a silicon target with first-harmonic laser radiation (λ = 1024 nm) at room temperature (27 °C) for 5 min. Each laser pulse ablates the target surface, generating silicon micro- and nanoparticles that are dispersed into the surrounding liquid. Upon contact with water, these particles undergo wet oxidation, which initiates at the outer surface and progressively leads to the formation of an SiO_2_–H_2_O colloid [35], as demonstrated by the following chemical equation:

(1) $$Si+2H_{2}O\to SiO_{2}+2H_{2}$$

The oxidation reaction is exothermic, and the high temperatures generated within the plasma plume during ablation further promote both silicon oxidation and particle fragmentation. However, after laser ablation of the Si target, micrometer-sized or larger Si particles that do not fully oxidize remain dispersed in the solution. To promote silicon fragmentation and further oxidation, the resulting colloidal suspension was subjected to an additional laser irradiation process, as schematically illustrated in Figure 1. A divergent lens is used to expand the diameter of the laser beam (second harmonic, λ = 532 nm) by approximately a factor of two (d = 1.4 cm). The pulse energy during irradiation was $E_{p}=50$ mJ, resulting in a fluence of $\Phi_{E}=200$ mJ·cm^−2^. The colloid was irradiated for 5 and 10 min under these conditions. To ensure uniform laser exposure, the suspension was continuously stirred using a magnetic stirrer.

The irradiation process induces cavitation effects that fragment the larger silicon particles generated during ablation, thereby promoting their oxidation. As demonstrated in the Results section of this research, this additional irradiation step significantly increases the concentration of nanometric silicon in colloidal suspension.

### 2.3. Chemical Process: Oxidation-Reduction Reactions

After preparing the colloidal suspension of nanometric silicon (SiO_2_-H_2_O colloid), 80 µL of a 50 mM aqueous AgNO_3_ solution was added to a total volume of 20 mL of the prepared colloid, resulting in a final Ag^+^ concentration of 0.2 mM.

Silver ions are reduced to metallic silver (Ag^0^) through the generation of free electrons in the solution resulting from silicon oxidation. The resulting silver atoms bond via metallic interactions to form silver nanoparticles, as described by the following chemical equation [36]:

(2) $$Si(NP)+4AgNO_{3}+2H_{2}O\to SiO_{2}(NP)+4Ag(NP)+4H^{+}+4NO_{3}^{-}$$

Ermakov et al. [26] proposed that the extremely high energies associated with laser ablation could result in the formation of multiple isomers of SiO_2_H_2_ on the surface of these SiO_2_ sub nanoparticles. The production and study of these isomers have been previously reported using electrical discharges [37]. Silver ions may be reduced through the reaction with these SiO_2_H_2_ isomers (Ag^+1^→Ag^0^), promoting the growth of silver nanoparticles [25].

When silver nanoparticles and SiO_2_ approach, their electron clouds interact, resulting a dipole moment due to the negative charge of the SiO_2_ (zeta potential ≈ −40 mV). This interaction promotes the formation of a SiO_2_ shell around the nanoparticles. The pH is neutralized, and the colloidal stability is improved by the addition of 0.1 mM of Na_2_CO_3_ at 0.1 mM to the colloid. Following the synthesis of Ag@SiO_2_ nanoparticles, aluminum chloride was incorporated into the colloidal solutions at final concentrations of 1 × 10^−4^ M, 1 × 10^−3^ M, and 2 × 10^−3^ M.

## 3. Results

### 3.1. Optical Properties of Colloidal SiO_2_ Before and After Adding Silver Ions

**Figure 2:**
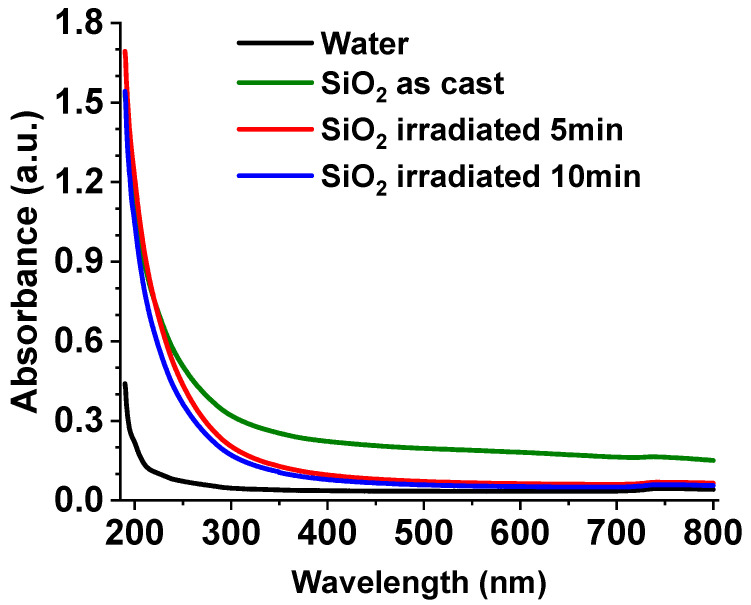
Absorption spectra of the as-cast SiO_2_ colloid, irradiated for 5 min, and irradiated for 10 min. The absorption spectrum of HPLC water was also added for reference.

The absorption spectra of the synthesized SiO_2_ colloids, both as-cast and after laser irradiation for 5 and 10 min, were recorded in the 200–800 nm range and are depicted in Figure 2.

Results show low absorption for all samples, as SiO_2_ is transparent down to approximately 200 nm [38]. The absorbance of the as-cast SiO_2_ colloid (green line) is high in the visible to near-infrared wavelength range. The behavior is believed to be the result of the scattering by particles in the solution that are comparable in size to the wavelength of the incident radiation.

During the irradiation process, micrometric and submicron-sized particles are detached by the laser-induced shockwave and subsequently oxidize upon contact with water. As a result, the absorption (scattering) is markedly reduced—particularly in the visible and near-infrared regions—as observed for the SiO_2_ colloid irradiated for 5 min (red line). This decrease is attributed to the fragmentation of larger silicon particles generated during the irradiation process [39]. When the irradiation time was increased to 10 min (blue line), the reduction in scattering compared to the 5 min sample was negligible. Therefore, all subsequent experiments were performed using samples irradiated for 5 min.

Using the Tauc method, the optical band gap values of the as-cast colloid and of the colloids irradiated for 5 and 10 min were estimated and the results are presented in Figure S6. The band gap values obtained for all samples lie in the range of approximately 6.13–6.24 eV, which is consistent with the presence of predominantly SiO_2_ in the colloids. Furthermore, the band gap increases with increasing irradiation time, suggesting that laser irradiation enhances the concentration of nanometric SiO_2_ within the colloidal suspension.

**Figure 3:**
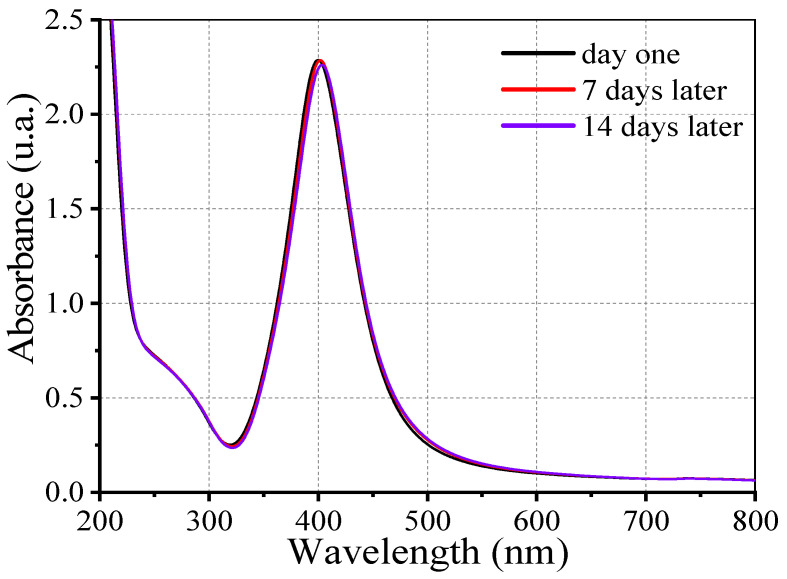
Absorption spectra of Ag@SiO_2_ NPs as a function of time, evaluated through 14 days. Ag@SiO_2_ NPs were synthesized using the irradiated SiO_2_–H_2_O colloid.

Figure 3 shows the UV–Vis absorption spectra of the synthesized Ag@SiO_2_ nanoparticles recorded immediately after synthesis, and after 7 and 14 days of aging. A distinct absorption band at ~400 nm is observed, which is characteristic of SPR of metallic silver nanoparticles.

Moreover, a secondary absorption feature of lower intensity appears at approximately 272 nm, which can be attributed to electronic transitions to higher-energy states within the quantum-confinement potential. The temporal stability of the plasmonic band (maximum intensity and FWHM) confirms that the nanoparticles maintain their structural and optical integrity under experimental conditions. This stability is attributed to the SiO_2_ shell that envelopes the nanoparticles, which provide colloidal stability.

The silica shell serves a formidable protective barrier that impedes oxidation, dissolution, and aggregation, thereby substantially reducing Ostwald ripening and maintaining the integrity of the nanoparticles over time.

In addition, this core–shell architecture enables the functionalization of targeted applications and enhances dispersion stability in media. In our analysis, a slight redshift in the SPR peak observed in UV–Vis spectra from 400 nm to 404 nm represents a minor increase in effective particle size or subtle particle interactions, which are typical signatures of early-stage Ostwald ripening or mild aggregation. The effectiveness of the silica shell in maintaining colloidal stability is demonstrated by the fact that the overall concentration of silver nanoparticles remains essentially constant, despite this redshift. However, colloidal stability of Ag@SiO_2_ nanoparticles is highly sensitive to multivalent electrolytes such as AlCl_3_, which induce rapid aggregation through double layer compression and charge neutralization by trivalent Al^3+^ cations, underscoring the importance of controlled ionic conditions for maintaining long-term dispersion stability.

### 3.2. Structural Properties of Colloids Measured Before and After Addition of Silver Ions Studied by X-Ray Diffraction (XRD)

**Figure 4:**
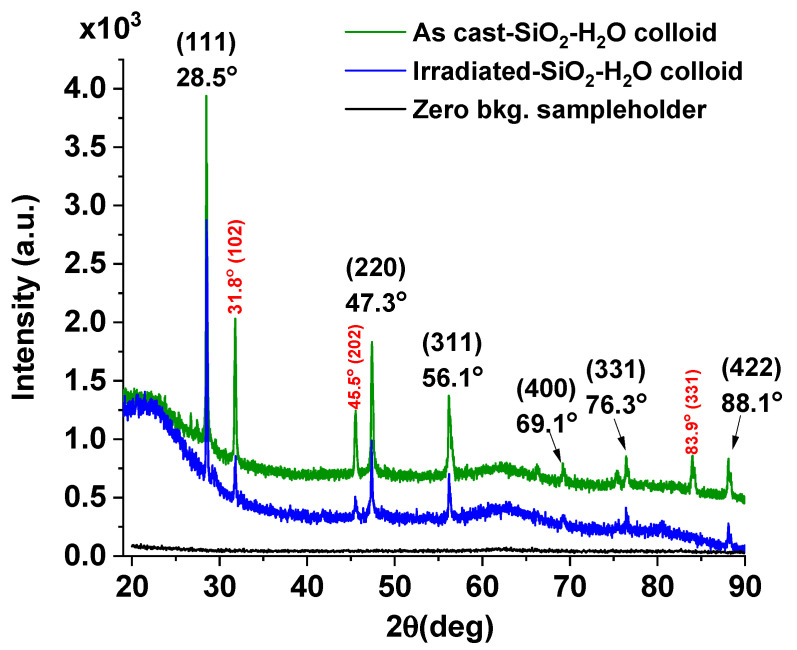
XRD patterns of colloidal SiO_2_–H_2_O obtained by laser ablation: as-cast colloid (green line) and irradiated colloid (blue line). For comparison, the diffractogram of the zero-background sample holder is also included.

Figure 4 shows the XRD patterns of the as-cast SiO_2_–H_2_O colloid (green line) and after irradiation (blue line).

In both cases, the diffractograms exhibit peaks at 28.5°, 47.3°, 56.1°, 76.3°, and 88.1°, corresponding to the (111), (220), (311), and (422) diffraction planes of cubic silicon (PDF 027-1402), respectively. The signals are narrow and display high overall intensity, indicating the long-range ordering characteristic of a crystalline material. This confirms the presence of crystalline silicon particles of micrometer or sub-micrometer size within the colloidal SiO_2_. These particles are ejected from the silicon target by the shockwave generated during laser ablation and do not fully oxidize to SiO_2_ due to their relatively large size.

The FWHM values of these silicon signals do not change significantly upon irradiation; however, their overall intensities are notably lower than those observed in the as-cast colloid.

Using Scherrer’s formula and the peak around 28°, crystallite sizes of 48 ± 1 nm and 53 ± 1 nm were calculated for the crystalline silicon, inside as-cast and irradiated colloid, respectively. The increase of about 10% in crystallite size of Si in the irradiated sample may be attributed to the additional energy absorbed by the material during the irradiation process. Figures S1 and S2 detail these calculations. For more information, see Supplementary Information (SI).

This increase in crystallite size should be reflected as an increase in the intensity of the Si signals in the irradiated colloid; however, the intensities decrease considerably. This behavior suggests that laser irradiation fragments the larger silicon particles into smaller ones, resulting in a substantial reduction in the concentration of crystalline silicon within the colloid. Once formed, these smaller particles undergo further oxidation, ultimately leading to the formation of amorphous SiO_2_ at the nanometer scale [40].

From XRD, it can be observed that the intensities of the diffraction signals corresponding to crystalline SiO_2_ are also markedly reduced after colloid irradiation. This observation is further supported by the decrease (75%) in integrated peak areas obtained from the fitting analysis, from 236 a.u. for the as-cast colloid to 60 a.u. for the irradiated colloid, which confirms that this phase also undergoes substantial fragmentation during colloid irradiation.

Furthermore, the diffractograms of both colloids exhibit narrow peaks at 31.8°, 45.5° and 83.9° corresponding to the (102), (202) and (331) diffraction planes of crystalline SiO_2_ (cristobalite, PDF 01-089-3434), respectively. The formation of this metastable SiO_2_ phase may be attributed to the high temperatures reached at the surface of the silicon target during the laser ablation process.

Using Scherrer’s formula and the diffraction peak at 31.8°, the crystallite sizes of crystalline SiO_2_ (Cristobalite) in the as-cast and irradiated colloids were estimated to be 41 ± 3 nm and 43 ± 3 nm, respectively. For more details, see Supplementary Information (Table S1).

Finally, it is important to highlight the low-intensity amorphous halo centered at 22°, which becomes more pronounced in the irradiated colloid and can be associated with the presence of amorphous SiO_2_ in the sample at this stage of the fabrication process.

**Figure 5:**
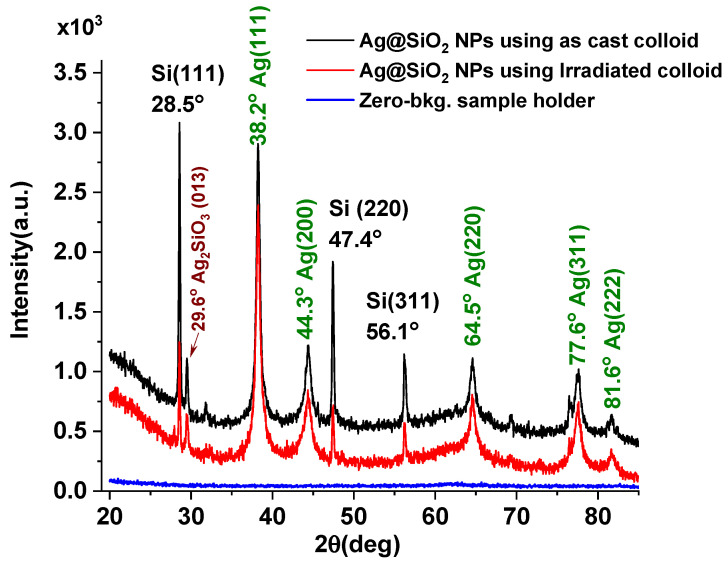
Diffraction pattern of Ag@SiO_2_ NPs synthesized using both the as-cast and irradiated colloid. For comparison, the diffractogram of the zero-background sample holder is also included.

Figure 5 depicts the results for X-ray diffraction (XRD) of the Ag@SiO_2_ NPs synthesized using both the SiO_2_-H_2_O as-cast and SiO_2_-H_2_O irradiated colloid.

In both cases, the diffractograms exhibit signals at 28.5°, 47.4°, and 56.1°, corresponding to the (111), (220), and (311) diffraction planes of cubic silicon (PDF 027-1402), respectively. These peaks are narrow and display high intensity, indicating the long-range ordering characteristic of a crystalline material, thereby confirming the presence of micrometer- or sub-micrometer-sized crystalline silicon particles within the colloid.

As observed from the diffractograms, the overall intensities and integrated peak areas of the silicon-related signals are reduced upon the addition of the silver salt. However, this reduction is more pronounced when the irradiated colloid is used for nanoparticle synthesis. This behavior indicates a considerable decrease in the concentration of crystalline silicon particles in the final dispersion containing the Ag@SiO_2_ nanoparticles.

Using Scherrer’s formula and the peak at 28.5°, crystallite sizes of 40 ± 1 nm and 45 ± 1 nm were calculated for crystalline silicon inside as-cast and irradiated colloid, respectively. Comparison with the values obtained before silver incorporation reveals that the crystallite size decreases by approximately 15% and 17% after adding the silver salt to the as-cast and irradiated SiO_2_–H_2_O colloid, respectively.

Consequently, the addition of silver salt to the SiO–H_2_O colloids is another factor contributing to the observed decrease in the XRD signals of crystalline Si. For more details, see Supplementary Information file (Figure S3).

Furthermore, diffractograms exhibit signals around 38.2°, 44.3°, 64.5°, 77.6° and 81.6° corresponding to the (111), (200), (220), (311) and (222) diffraction planes of the Ag FCC crystalline structure, in good agreement with the reference pattern JCPDS 00-004-0783. Both the FWHM and the signal intensities are consistent with the presence of nanoscale material. For more details, see Supplementary Information file (Figure S4 and Table S2).

For calculating the lattice parameter and crystallite size of the Ag@SiO_2_ NPs, the most intense reflection (111) was used. Deconvolution of these signals required two Gaussian components to differentiate the contribution of the smaller particles (<10 nm), which dominate the size distribution according to TEM analysis, from that of the larger ones (>10 nm). Since the XRD peak intensity is proportional to the scattering volume, which, for spherical nanoparticles, scales with the cube of the particle diameter, the scattering from a 20 nm particle is 64 times stronger than that from a 5 nm particle (20^3^/5^3^ = 64). Consequently, even a small fraction of larger particles in the size distribution can overshadow the contribution of the far more abundant smaller ones. Similar results were reported by Ermakov et al. [26].

To estimate the mean crystallite size, Scherrer’s formula was applied, yielding values of 20 ± 2 nm for the narrower Gaussian peak and 7 ± 1 nm for the broader one. These values represent the Ag@SiO_2_ nanoparticles core diameter and are similar whether the nanoparticles are fabricated using the as-cast or the laser-irradiated colloid.

The lattice parameter calculated from the XRD pattern is a = 4.07318 Å, in good agreement with the literature value a = 4.086 Å.

The diffraction signal at 29.6° (2θ) may correspond to the (013) plane of monoclinic silver silicate (Ag_2_SiO_3_, PDF 76-2088). This phase could act as an intermediate during the formation of the core–shell structure. The low intensity observed in the diffractogram, suggests only a scarce presence of this phase in the colloid.

It is important to highlight the low-intensity amorphous halo centered at 22°, present in samples containing Ag@SiO_2_ NPs synthesized using both the SiO_2_ as-cast and SiO_2_ irradiated colloid, which can be associated with the presence of amorphous SiO_2_ in the sample.

Finally, XRD results reveal that the cristobalite signals at 31.8°, 45.5°, and 83.9°, which are present in both the as-cast and irradiated colloids, nearly vanished once the silver salt was added, indicating that no crystalline SiO_2_ is present in the final Ag@SiO_2_ nanoparticle colloids. Most authors who use this fabrication technique have reported the same result [25,26,27]; however, in those studies, the colloid prior to the addition of the silver salt was not analyzed, and consequently the origin of the amorphization or loss of crystallinity in both crystalline SiO_2_ and crystalline Si was not addressed.

Silver has been experimentally shown to induce preferential crystallization and orientation in doped tin sulfide (SnS) systems, primarily through dynamic regulation of chemical species during synthesis or irradiation. In Ag-doped SnS, silver ions and atoms act as nucleation centers and modify the local chemical environment, promoting the orientation and growth of specific crystallographic planes. This effect arises from fluctuations in reductive and oxidative species, with Ag modulating redox cycles to enable controlled crystal growth with preferred orientation. The established role of Ag in modifying nucleation kinetics and directing crystallinity suggests a plausible influence on cristobalite (SiO_2_), where nucleation-growth dynamics indicate that Ag may affect morphology and crystalline orientation. Experimental evidence from XRD and morphology confirms Ag’s role as a nucleation modulator, supporting its potential for tailored silicate synthesis in advanced materials. Observed changes in XRD patterns, such as preferred orientation and intensity, are attributed to Ag incorporation, highlighting a pathway to explore metallic cores as crystallization modulators in silicate materials for controlled synthesis [41].

Sanal et al. [42] reported the growth of silver nanoparticles in SiO_2_ matrix by the co-sputtering technique. Silver nanoparticles are uniformly dispersed within the silica matrix, and their volume fraction increases with sputtering time, while particle size remains nearly constant. This morphology strongly suggests that Ag atoms condense first during the processing procedure, forming discrete metallic clusters that act as preferential nucleation centers for SiOx species. This behavior was attributed to the high surface energy and chemical activity of Ag, combined with numerous dangling bonds on both Ag surfaces and silica pore walls, which promote intensive interfacial interactions. These interactions include Ag-Si chemical bonding and electron transfer from Ag to SiO_2_, mechanisms that minimize interfacial energy and stabilize the composite structure. The presence of dangling bonds at Ag surfaces and silica pore walls could be proposed as the origin of strong interfacial interactions and can be indirectly confirmed by FTIR in similar systems. Dangling bonds on silica typically terminate as hydroxyl groups (Si–OH), producing characteristic absorption near ~3400 cm^−1^ (O–H stretching) and ~950 cm^−1^ (Si–OH bending). The high intensity of these bands could indicate a large number of unsaturated Si sites. As can be seen from FTIR Si-O-Si region, a large amount of Si sites could be related to the broadened and intense band (1250–1000 cm^−1^).

When silica is irradiated, this site’s availability decreases in intensity, suggesting that laser input fragments the silica, allowing silver ions to be covered to further compose the core–shell nanostructure. This is observed in the FTIR of Ag@SiO_2_ (using irradiated colloid) where the Si-O-Si vibration mode slightly decreases in intensity, indicating that Ag nucleation sites are now occupied by silica molecules.

Additionally, the Si–O–Si asymmetric stretching band (~1080 cm^−1^) exhibits reduced intensity which can be attributed to structural distortions arising from Ag–Si interfacial interactions, such as Ag–Si bonding or electron transfer effects. Therefore, FTIR provides indirect evidence of dangling bonds by detecting hydroxyl terminations and network perturbations, supporting the hypothesis that these reactive sites drive nucleation and interfacial coupling in Ag–SiO_2_ nanocomposites. For more details, see Supplementary Information (Figure S5).

Using the Williamson–Hall (W–H) approach, the microstrain (ε) and dislocation density (δ) were calculated for both as-cast and laser-irradiated SiO_2_–H_2_O colloids, as well as for the corresponding solutions after silver incorporation. The analysis was performed using the high-intensity diffraction peaks of silver, namely (111), (200), (220), and (311) together with the dominant peaks associated with crystalline Si and SiO_2_. For these calculations, the following equation was used:

(3) $$\beta Cos\theta =\varepsilon \left(4Sin\theta \right)+\frac{K\lambda }{D}$$

The values of full width at half maximum (FWHM, *β*) were measured for each peak within the X-ray diffraction pattern. The corresponding microstrain (*ε*) contributions were considered. The Bragg angle (*θ*), together with the shape factor (*K*) and the X-ray wavelength (*λ*), was used to determine the structural parameters. The results are summarized in Table S3.

The Williamson–Hall (W–H) method could provide complementary evidence for the nucleation role of silver in Ag-SiO_2_ nanocomposites. W–H separates peak broadening contributions from crystallite size and microstrain in XRD patterns. If applied to Ag diffraction planes, the analysis would be expected to show significant lattice strain. High microstrain reflects strong interfacial interactions between Ag clusters and the amorphous SiO_2_ matrix. These strain effects arise from lattice mismatch and chemical bonding at the interface, consistent with the mechanism where Ag acts as preferential nucleation sites for SiOx species. Laser irradiation of the SiO_2_–H_2_O colloid produces predominantly amorphous or poorly crystalline material. The incorporation of Ag further enhances this effect, suppressing the cristobalite signals in the XRD diffractograms due to rapid fragmentation, high dislocation density, and increased nucleation. Thus, W–H analysis could reinforce the interpretation that silver initiates nucleation and stabilizes the core–shell structure through interfacial coupling. For more details, see Supplementary Information (Table S3).

From our findings, we observe that an increase in microstrain accompanied by a reduction in dislocation density promotes more ordered crystal growth of the Ag cores. In contrast, pure SiO_2_ exhibits increased microstrain and higher dislocation density, suggesting the role of silver in modulating the structural evolution of silica toward a more amorphous phase [43].

### 3.3. Electrophoretic Properties and pH Measurements of SiO_2_-H_2_O Colloids and Ag@SiO_2_ NPs Synthesized Using the Irradiated SiO_2_–H_2_O Colloid

**Figure 6:**
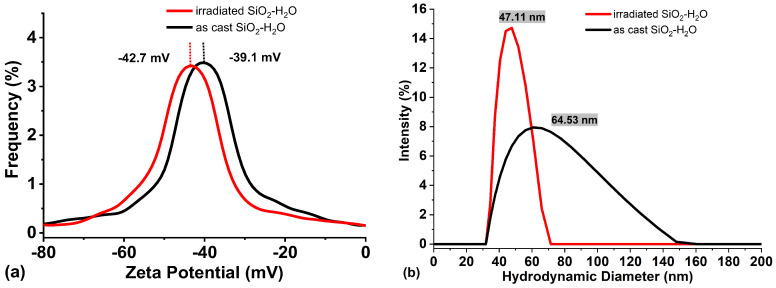
(**a**) Zeta potential (mV) and (**b**) hydrodynamic diameter of SiO_2_ colloid as cast (black line) and irradiated by 5 min (red line).

Zeta potential measurements of the as-cast SiO_2_-H_2_O colloid and after irradiation are presented in Figure 6a. The average zeta potential of the as-cast colloid is approximately –39 mV, shifting slightly to more negative values (–42 mV) after irradiation. This change suggests that laser irradiation generates smaller silicon particles that oxidize more rapidly, thereby increasing the concentration of nanometric SiO_2_ in the colloid, which in turn increases the absolute value of the sample’s zeta potential [44]. An increase in the SiO_2_ concentration can also enhance the colloid’s efficiency in reducing metal ions such as Ag^+^, Au^+^, Cu^+^, and others. It is important to note that the zeta potential is widely regarded as a reliable indicator of colloidal stability, and colloids with zeta potential values exceeding ±30 mV are considered stable [45].

The hydrodynamic diameter results are depicted in Figure 6b and reveal that, upon irradiation, the mean particle size decreases from 64 to 47 nm, accompanied by an increase in the signal intensity. This behavior indicates a higher concentration of smaller nanoparticles within the colloid, further supporting the fragmentation of larger Si particles.

Furthermore, a significant reduction in the full width at half maximum (FWHM) of the size distribution is observed for the irradiated colloid (red line), indicating that the fragmentation of larger particles results in a narrower nanoparticle size distribution. It is important to note that the hydrodynamic diameter does not correspond to the actual particle size, but rather to the apparent size of the particles in solution, including their solvation layer.

Table 1 presents pH measurements of SiO_2_–H_2_O colloids over an 8-day period. A noticeable increase in pH (from 6.18 to 7.10 and 7.54) is observed during the initial days for both samples. The release of electrons (e^−^) into the medium is the consequence of the oxidation of Si particles, which ultimately leads to an increase in pH. Once the oxidation of silicon is complete, the pH stabilizes and remains relatively constant for both samples. Minor fluctuations observed in the later stages are attributed to the exposure of atmospheric CO_2_.

The results indicate that the irradiation process does not exert a significant effect on the pH of the SiO_2_–H_2_O colloid, suggesting that irradiation does not substantially alter the chemical dynamics of the system under the experimental conditions employed. It is also worth noting that the neutral pH of the colloidal solution and the electron liberation to the medium make it ideal for metal reduction synthesis.

### 3.4. Electrophoretic Properties and pH Measurements of Ag@SiO_2_ NPs Synthesized Using the Irradiated SiO_2_–H_2_O Colloid

**Figure 7:**
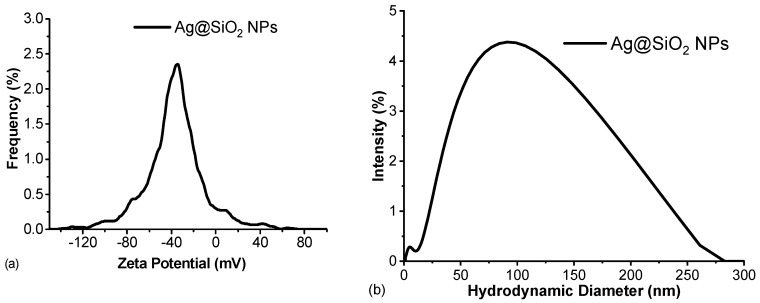
(**a**) Zeta potential and (**b**) hydrodynamic diameter graphs of Ag@SiO_2_ NPs synthesized using the irradiated SiO_2_–H_2_O colloid.

Figure 7 depicts the zeta potential and hydrodynamic diameter of the synthesized Ag@SiO_2_ nanoparticles. An aliquot of the undiluted NPs solution was placed in the Omega cuvette to carry out the measurements.

The measured zeta potential of −35.16 mV falls within the typical range of stable colloidal dispersions. When compared to the SiO_2_ extracted in the initial stage of the synthesis (ζ ≈ −43 mV), the Ag@SiO_2_ NPs display a slightly lower magnitude of ζ. This observation would suggest that the SiO_2_ employed to reduce the metal salt is the primary factor controlling the high surface charge of the Ag@SiO_2_ NPs. Consistent with the HRTEM measurements both in this work and previous report [12], the PLD process effectively produces Ag nanoparticles coated with a stoichiometric SiO_2_ shell of approximately 2–3 nm in thickness.

Moreover, this near-neutral pH value of 7.6, which is significantly above the isoelectric point of SiO_2_ (pI = 2–3), is a critical parameter contributing to colloidal stability. At this pH, surface silanol groups (≡Si-OH → ≡Si-O^−^) on the silica surface are partially deprotonated, generating a negative surface charge (zeta potential −39 to −42 mV) that provides sufficient electrostatic repulsion limiting Ag@SiO_2_ aggregation (zeta potential −35 mV). This has been validated by TEM showing individual nanoparticles without aggregates. Deviations from this pH, as well as changes in the ionic strength or composition of the medium, can have a substantial influence on the zeta potential, resulting in the destabilization of the colloidal system.

The relatively large hydrodynamic diameter observed (~91 nm) can be attributed to the solvation layer surrounding the negatively charged Ag@SiO_2_ nanoparticles. This layer, which is composed of the solvent molecules and ions that are tightly bound, extends beyond the nanoparticle core, thereby increasing the apparent particle size as demonstrated by dynamic light scattering. The hydrodynamic diameter and colloidal behavior of the system are significantly influenced by the formation of this electrical double layer, which is influenced by electrostatic interactions with ions in the surrounding medium. The colloidal stability of the system is strongly supported by the measurements of both the zeta potential and hydrodynamic diameter. The high absolute value of the zeta potential (35.16 mV) indicates that the Ag@SiO_2_ nanoparticles are effectively stabilized against aggregation by electrostatic repulsion. This stability is further supported by the observation that their hydrodynamic diameter remains constant over time.

The UV–Vis spectroscopy analysis further supports these findings, as it demonstrates that the characteristic absorption band of the nanoparticles persists throughout the study.

The mean values of pH, zeta potential, and hydrodynamic diameter obtained for the Ag@SiO_2_ nanoparticles under the experimental conditions confirm the correlation between these parameters and the stability of the system.

### 3.5. TEM and HRTEM of Ag@SiO_2_ NPs Processed Using the SiO_2_-H_2_O Irradiated Colloid

**Figure 8:**
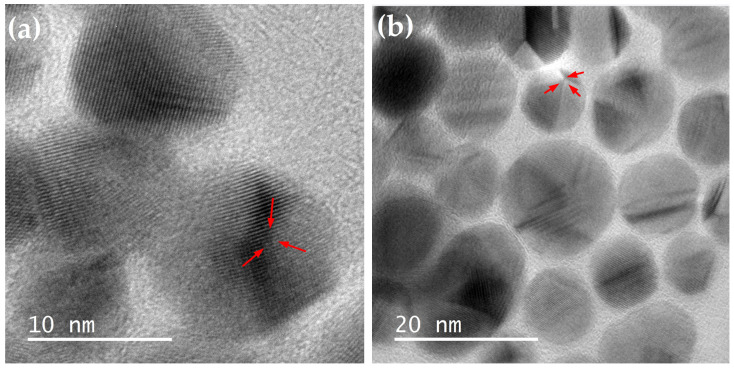

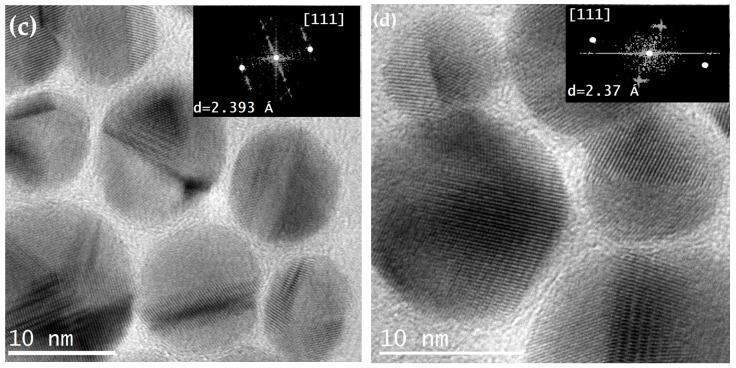
(**a**) High-resolution image and (**b**) low-resolution image of Ag@SiO_2_ nanoparticles. The red arrows observed in the images show twin planes characteristic of AgNPs. (**c**,**d**) are HRTEM images of Ag@SiO_2_ nanoparticles. The inset in the micrographs are the FFT of the whole image showing the (111) direction of the FCC silver structure.

The morphological and structural properties of the synthesized Ag@SiO_2_ nanoparticles (NPs) were evaluated using Transmission Electron Microscopy (TEM), and the micrographs were subsequently processed with Digital Micrograph software 3.10.1 (Gatan Inc., Pleasanton, CA, USA). High-resolution TEM (HRTEM) images reveal homogeneously spherical silver nanoparticles with a well-defined crystalline structure, encapsulated by an amorphous SiO_2_ shell that effectively prevents particle agglomeration. A typical feature of Ag nanoparticles is the presence of twin planes (highlighted by red arrows in Figure 8a,b), which arise when two or more families of crystallographic planes exhibit symmetrical orientation.

The insets in Figure 8c,d show the Fast Fourier Transform (FFT) analyses of the HRTEM images of the Ag@SiO_2_ NPs. From these analyses, the measured interplanar spacings (2.39 and 2.37 Å) are in good agreement with the interplanar distance of the (111) crystallographic plane of face-centered cubic (FCC) silver (d = 2.36 Å), as reported in the PDF card 04-0783.

**Figure 9:**
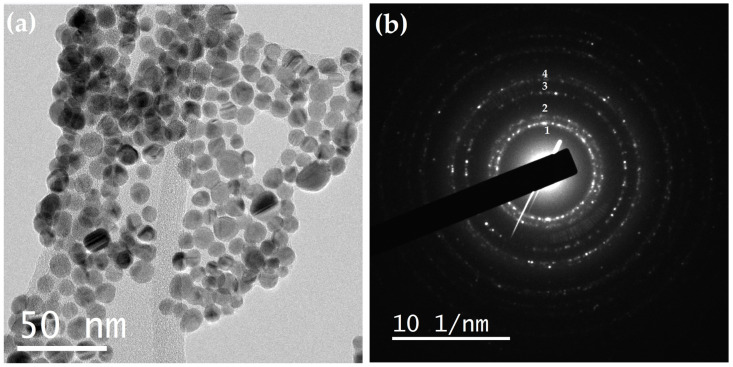
(**a**) TEM image and (**b**) ring diffraction pattern of Ag@SiO_2_ NPs.

Notably, despite the high nanoparticle density, no evidence of coalescence is observed (Figure 9a), demonstrating the effectiveness of the SiO_2_ coating in maintaining particle separation. The corresponding selected-area electron diffraction (SAED) pattern is shown in Figure 9b. From this pattern, the interplanar spacings were calculated and compared with the values reported for Ag in the reference card JCPDS 04-0783 (see Table 2).

**Figure 10:**
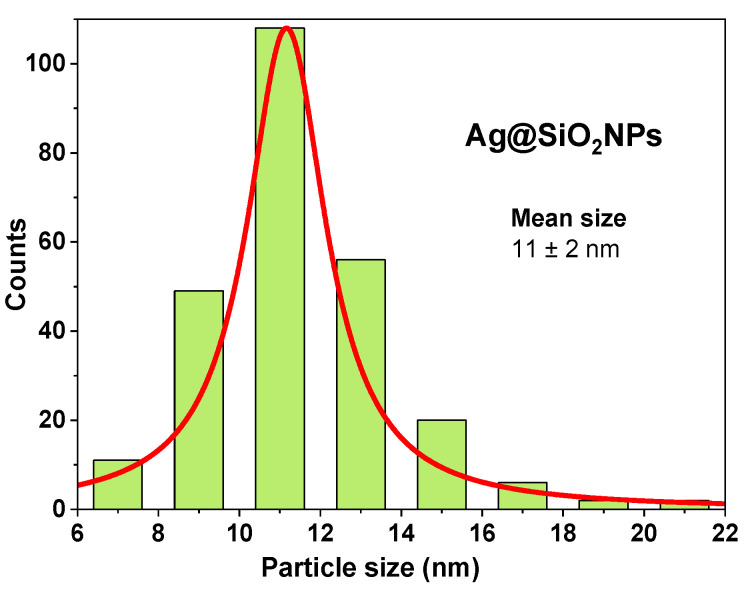
Size distribution histogram and mean size of Ag@SiO_2_ NPs.

The Ag@SiO_2_ NPs histogram (Figure 10) obtained from the TEM images reveals a size distribution (from 6 to 22 nm) with a mean size of 11 ± 2 nm, which coincides with XRD results (reported above), and other values reported in the literature for Ag@SiO_2_ NPs synthesized by this method [27,36].

#### Formation Mechanism of Ag@SiO_2_

The synthesis of silver-silica core–shell nanoparticles via PLAL proceeds through a sequence of physical and chemical processes rather than a conventional chemical reaction. Upon irradiation of the target with nanosecond laser pulses in a colloidal suspension of mesoporous silica, an intense plasma plume is generated at the solid–liquid interface. This plasma reaches extreme thermodynamic conditions (temperatures of several thousand Kelvin and pressures in the order of tens of GPa), leading to material ejection and cavitation bubble formation. The high-energy environment partially disrupts the silica network, releasing SiO_2_ fragments and silanol groups (Si–OH) into the liquid phase.

These species condense onto the surface of pre-existing silver nanoparticles, forming a silica shell whose thickness depends on the structural integrity of the starting silica material. The resulting core–shell architecture enhances colloidal stability without the need for surfactants. The overall process can be summarized as:

(4) $$\mathrm{Si}\;(\mathrm{solid})\overset{\mathrm{laser ablation}}{\to }\mathrm{Si}(\mathrm{fragments})+{\mathrm{SiO}}_{2}\;\overset{\mathrm{laser irradiation}}{\to }{\mathrm{SiO}}_{2}\;{\mathrm{SiO}}_{2}\;+{Ag}^{+}⟶{\mathrm{Ag}@\mathrm{SiO}}_{2}\left(\mathrm{core–shell}\right)$$

### 3.6. Effect of AlCl_3_ on the Surface Charge and Aggregation of Ag@SiO_2_ NPs

Ag@SiO_2_ NPs were synthesized using the irradiated SiO_2_–H_2_O colloid and subsequently diluted 1:10 with HPLC-grade water to better observe the effect of Al addition. To modulate the surface charge of the Ag@SiO_2_ nanoparticles, aluminum chloride (AlCl_3_) was added to the colloidal suspension at varying concentrations. Details of the sample preparation and experimental conditions are summarized in Table 3.

The Al^3+^ ions introduced by AlCl_3_ act as multivalent cations that effectively screen and neutralize the negatively charged Si-OH groups on the nanoparticle surface. The electrostatic repulsion between nanoparticles is reduced by this neutralization, which compresses the electrical double layer surrounding each nanoparticle; this destabilizes the colloidal system and promotes agglomeration [46]. Furthermore, Al^3+^ ions can function as bridging agents, allowing for the formation of ionic cross-links between nanoparticle surfaces, thereby enhancing agglomeration. The balance between repulsive electrostatic forces and attractive van der Waals and ion-bridging interactions governs the extent and kinetic of agglomeration. Over a five-day period, the optical and electrophoretic properties of the suspensions were monitored to assess the influence of AlCl_3_ concentration on the aggregation behavior and stability of the nanoparticles. At controlled low concentrations, silica atoms can be replaced with aluminum ions to allow for enhanced stability by creating a modified surface that provides superior electrostatic stabilization in comparison to the pure silica system. The stabilization mechanism operates through surface modification rather than double layer compression. When aluminum is incorporated into the silica surface, it creates negatively charged aluminosilicate sites that enhance particle repulsion and extend the effective range of electrostatic stabilization [47].

#### 3.6.1. Optical Properties of Samples Containing Ag@SiO_2_ NPs + AlCl_3_

**Figure 11:**
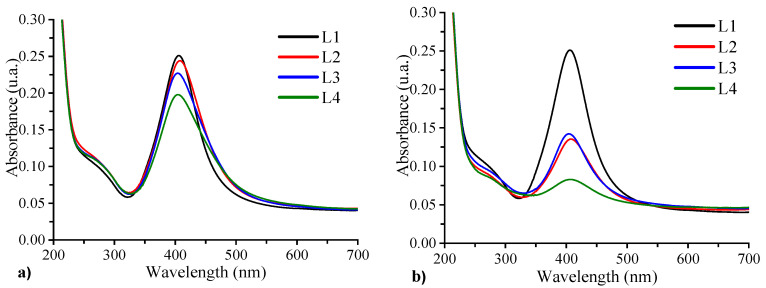
Absorption spectra of L1, L2, L3, and L4 samples evaluated on the 1st (**a**) and the 5th day (**b**).

Figure 11a,b show the UV–Vis absorption spectra of samples L1, L2, L3, and L4 recorded on the first and fifth days after preparation. On day one, all samples exhibit a surface plasmon resonance (SPR) band centered near 400 nm. The results indicate that increasing the Al concentration leads to a decrease in plasmon intensity accompanied by a broadening of the full width at half maximum (FWHM). This effect is most pronounced for sample L4, which contains the highest Al concentration.

The primary cause of this behavior is the role of the Al^3+^ ions in mediating nanoparticle aggregation. Aluminum ions, which are trivalent cations with a high charge density, effectively screen and neutralize the negative surface charge provided by the silanol groups on the SiO_2_ shell of the nanoparticles. Al^3+^ ions promote aggregation and facilitate closer nanoparticle approach by compressing the electrical double layer and reducing electrostatic repulsion. Furthermore, Al^3+^ ions can function as bridging species, establishing ionic cross-links between neighboring nanoparticles and stabilizing larger aggregates that absorb light at longer wavelengths, as can be seen in the absorption spectra. Simultaneously, the decrease in absorption intensity is indicative of a reduction in the population of small, dispersed nanoparticles, which typically absorb near 400 nm because of the agglomeration into larger clusters, which influences optical properties. The significant decrease in intensity observed in sample L4 when contrasted with L2 and L3 is the result of its elevated AlCl_3_ concentration (2 × 10^−3^ M). The absence of a second absorption band at longer wavelengths likely indicates that the concentration of aggregated nanoparticles remaining dispersed is below the UV–Vis detection threshold, consistent with the relatively low initial nanoparticle concentration of 10%.

After 5 days of sample preparation (Figure 11b), the absorption band intensity decreases, indicating ongoing Al^3+^-induced nanoparticle aggregation and sedimentation. This process results in precipitation and a corresponding reduction in the concentration of suspended nanoparticles. In contrast, sample L1, which lacks AlCl_3_, maintains stable absorption features with no significant spectral changes, confirming the stability of the unmodified nanoparticles.

#### 3.6.2. Zeta Potential and Hydrodynamic Diameter

**Figure 12:**
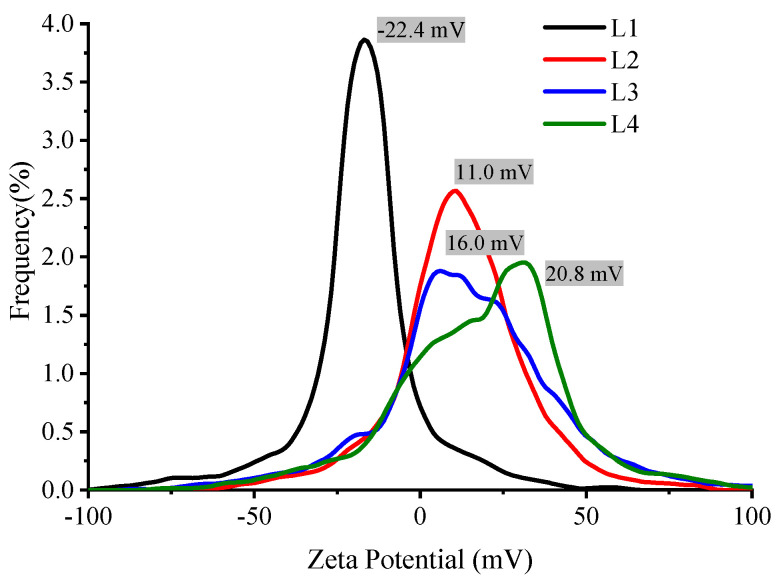
Zeta potential of Ag@SiO_2_ NPs with different concentrations of AlCl_3_.

According to the theory of colloids, zeta potential is a function of the electrolyte concentration and the ion valence. This is reflected in the results shown in Figure 12, where a decrease in the zeta potential is observed as a function of AlCl_3_ concentration since Al^+3^ ions counteract the negative charge of the SiO_2_ shell. This reduces the colloidal repulsive potential, thereby facilitating the aggregation of Ag@SiO_2_ NPs.

**Figure 13:**
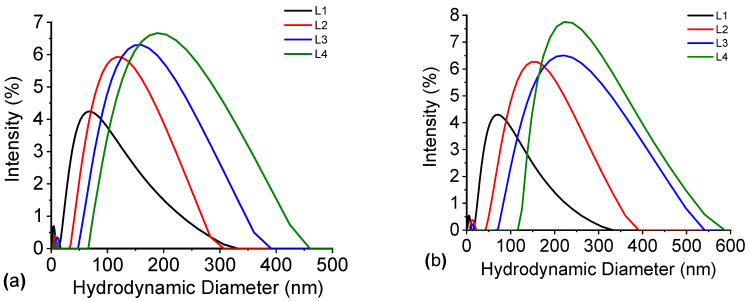
Hydrodynamic diameter of samples L1, L2, L3, and L4 evaluated: (**a**) the 1st day and (**b**) the 5th day.

Figure 13 depicts the time evolution of the hydrodynamic diameter of the samples. The results demonstrate significant differences among the samples when measured on the first day (Figure 13a) and the fifth day (Figure 13b).

The hydrodynamic diameter of the Al-free sample (L1) remains nearly constant throughout the evaluated period, indicating good colloidal stability. In contrast, all samples containing AlCl_3_ exhibit a clear increase in particle size, with the most pronounced growth observed in the sample with the highest AlCl_3_ concentration (green line).

The graphs in Figure 13b also illustrate that the Ag@SiO_2_ nanoparticles have the potential to form larger aggregates as storage time increases. This behavior is attributed to Al^3+^ ions, which screen the negative surface charge of the silica solvation shell, thereby reducing electrostatic repulsion and promoting nanoparticle aggregation. Therefore, it can be concluded that a concentration of 2 × 10^−3^ M of AlCl_3_ is sufficient to cause the agglomeration and surface charge modification of nanoparticles. Table 4 summarizes the hydrodynamic diameter and PDI of samples evaluated at day 1 and day 5.

### 3.7. Isoelectric Point and pH Measurements

**Figure 14:**
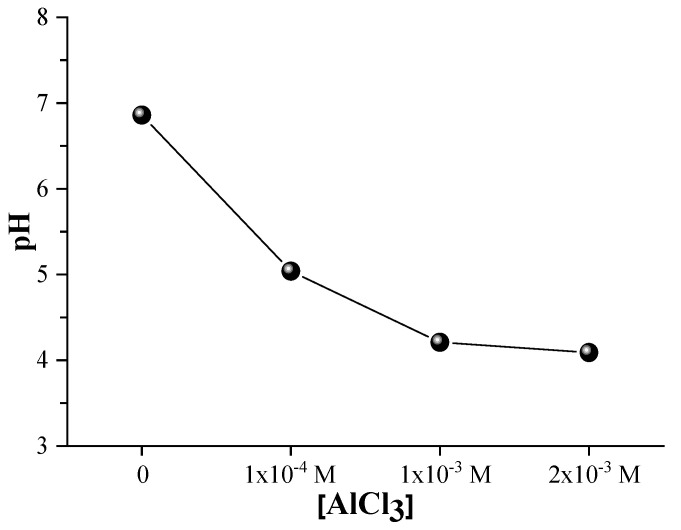
pH measurements of Ag@SiO_2_ NPs with different concentrations of AlCl_3_.

The colloidal stability of the Ag@SiO_2_ nanoparticles is substantiated by the fact that the sample maintains a neutral pH (approximately 7) as depicted in Figure 14. The pH gradually decreases as the concentration of AlCl_3_ increases, resulting in a more acidic environment. This acidification results from the hydrolysis of dissolved AlCl_3_, which releases Al^+3^ ions. Water and hydroxide ions react with these ions to form Al (OH)_3_, which releases protons (H^+^) and increases the hydrogen ion concentration ([H^+^]), thereby lowering the pH of the solution, according to:

(5) Al^3+^ + 3H_2_O → Al (OH)_3_ + 3H^+^

Nanoparticles’ microbicidal activity is significantly influenced by the acidic medium generated by AlCl_3_. The release of silver ions from the silver core is primarily responsible for antimicrobial properties, which is enhanced by a lower pH. The bactericidal mechanisms of silver ions are multifaceted: they disrupt the integrity of cell membranes, generate reactive oxygen species that provoke oxidative damage, bind to thiol groups in proteins and DNA, thereby inhibiting vital functions, and interfere with replication processes, resulting in eventual cell death. The controlled release of silver ions may be facilitated by a partial dissolution or restructuring of the silica shell, which is also promoted by Al^+3^ ions. Meanwhile, Al^+^^3^ ions also facilitate nanoparticles aggregation by neutralizing surface charges, which can elevate localized silver ion concentrations and improve microbicidal efficacy. Thus, the nanoparticles’ microbicidal efficacy is synergistically enhanced by the combined effects of AlCl_3_-induced acidification and surface charge modification. To optimize antimicrobial performance and maintain colloidal stability, it is essential to achieve an optimal balance of pH and nanoparticle aggregation.

**Figure 15:**
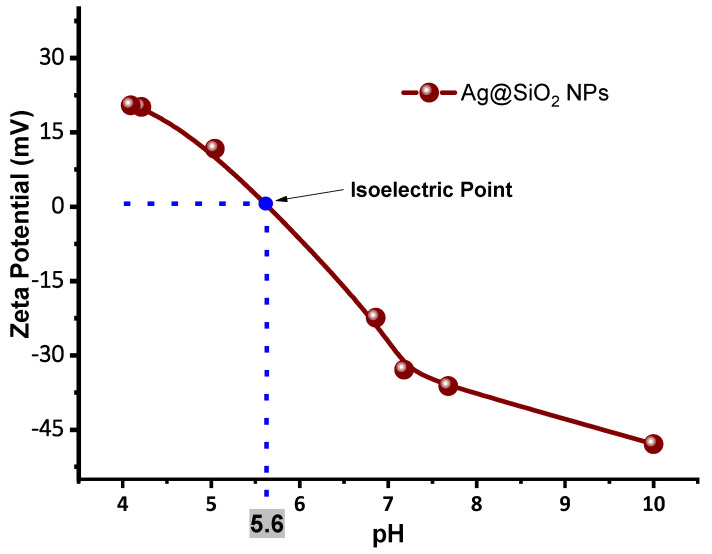
Zeta potential of Ag@SiO_2_ NPs as a function of pH, showing its isoelectric point.

Since the hydrogen ion concentration affects the solvation layer surrounding the nanoparticles, the pH is directly related to zeta potential and plays a crucial role in colloidal stability. Figure 15 shows a graph of the zeta potential as a function of the pH at which the Ag@SiO_2_ nanoparticles are presented, highlighting the isoelectric point (IEP), which is the pH at which a particle has a net charge of zero, and electrostatic repulsion is minimal. In this system, the IEP is approximately 5.6, which indicates the threshold at which aggregation initiates as a result of the loss of surface charge stabilization. This behavior is significantly influenced by the concentration of trivalent aluminum ions, which compress the electrical double layer and screen the negative charges on the silica surface, thereby reducing the magnitude of the zeta potential. The efficacy of this charge neutralization is significantly influenced by the concentration of the ions in relation to the Debye length, which is the characteristic distance by which the electrostatic interactions are screened in the medium [48].

The Debye lengths for AlCl_3_ concentrations (1 × 10^−4^ M, 1 × 10^−3^ M, and 2 × 10^−3^ M) that were examined are approximately 12.4 nm, 3.9 nm, and 2.8 nm, respectively. The most optimal condition is achieved by the concentration of 1 × 10^−3^ M as the hydrodynamic diameters of Ag@SiO_2_ nanoparticles are on the order of 10–20 nm from UV–Vis analysis. Achieving optimal particle aggregation without rapid precipitation is facilitated by this concentration, which induces effective but controlled double-layer compression, since the particle size is comparable to the Debye length.

A larger Debye length is the result of a lower concentration (1 × 10^−4^ M) which is sufficient for strong charge screening and particle interaction. This lower concentration maintains high colloidal stability but potentially could limit microbicidal efficacy due to reduced silver ions release. On the other hand, the double layer undergoes excessive compression at the highest concentration (2 × 10^−3^ M), resulting in rapid and widespread aggregation that may result in sedimentation and the loss of nanoparticle functionality.

Thus, it is imperative to achieve a balance between effective aggregation and colloidal stability by optimizing the modulation of surface charge and zeta potential with Al^+^^3^ ions that are located near the Debye length that corresponds to nanoparticle size. This balance enhances microbicidal performance by fostering increased silver ions release and facilitating nanoparticle–microbe interactions while preventing premature nanoparticle loss.

Thus, we can conclude that a colloid of Ag@SiO_2_ NPs with a pH ≈ 5.6 will have zero charge, marking the point at which aggregation begins.

## 4. Conclusions

X-Ray diffraction, dynamic and electrophoretic scattering, and UV–Vis spectroscopy provide physical and chemical evidence that the irradiation of the SiO_2_ colloid at 532 nm and an energy density of 200 mJ/cm^2^ efficiently fragments micrometer-sized or larger Si particles produced from the laser shockwave that do not fully oxidize and remain dispersed in the solution. Laser irradiation of SiO_2_-H_2_O colloids decreases the overall XRD intensities of both crystalline Si and crystalline SiO_2_ (cristobalite) signals in the colloid, suggesting this process produces predominantly amorphous or poorly crystalline material. After adding the silver salt, a decrease in crystallite size of 15–17% is observed in Si signals, and the signals for crystalline SiO_2_ almost vanished, suggesting the role of silver in modulating the structural evolution of silica toward a more amorphous phase.

According to XRD results, the core diameters of the Ag@SiO_2_ nanoparticles are similar whether the nanoparticles are fabricated using the as-cast or the laser-irradiated colloid.

The SiO_2_ colloid exhibits a high zeta potential (−42 mV), supporting the formation of a silica shell around Ag nanoparticles and the successful synthesis of Ag@SiO_2_ NPs. The laser-synthesized SiO_2_ colloid provides stability, surface charge, and effective isolation of the metallic core, reducing its reactivity. Zeta potential measurements show that low concentrations of AlCl_3_ promote the aggregation by modifying pH and surface charge, reaching +20.8 mV at a 2 × 10^−3^ M. This positively charged aggregation may favor microbicidal activity by enhancing adhesion to negatively charged microbicidal membranes. Increasing AlCl_3_ concentration reduces the Debye length due to higher ionic strength, compressing the electrical double layer, increasing hydrodynamic diameter, and promoting aggregation. It is important to note that higher zeta potential is associated with enhanced colloidal stability, which is essential for the proper dispersion of nanoparticles and the optimization of their microbicidal efficacy. In the design and application of AgNPs as effective antimicrobial agents, the significance of controlling ionic strength and surface charge is emphasized by these findings. The isoelectric point (the pH value at which particles have a net charge of zero) for the Ag@SiO_2_ NPs is observed at a pH of 5.6 value, at which the aggregation process may begin to occur.

## Figures and Tables

**Table 1:** Values of pH measurements over time (days).

Sample	Day 1	Day 4	Day 5	Day 6	Day 7	Day 8
SiO_2_–H_2_O as cast	6.18	7.54	7.12	7.00	6.82	6.80
SiO_2_–H_2_O irradiated (5 min)	6.18	7.10	7.10	7.04	6.88	6.88

**Table 2:** Ring diffraction pattern analysis.

Ring Number	Diffraction Plane	Calculated Distance [Å]	PDF Distance [Å]	Error (%)
1	1 1 1	2.40	2.36	4
2	2 0 0	2.08	2.04	4
3	2 2 0	1.50	1.45	5
4	3 1 1	1.24	1.23	1

**Table 3:** Summary of the Ag@SiO_2_ NPs + AlCl_3_ samples evaluated.

ID Sample	Sample Content
L1	H_2_O HPLC + Ag@SiO_2_ NPs 10% (20 mL)
L2	H_2_O HPLC + Ag@SiO_2_ NPs 10% + 1 × 10^−4^ M AlCl_3_ (20 mL)
L3	H_2_O HPLC + Ag@SiO_2_ NPs 10% + 1 × 10^−3^ M AlCl_3_ (20 mL)
L4	H_2_O HPLC + Ag@SiO_2_ NPs 10% + 2 × 10^−3^ M AlCl_3_ (20 mL)

**Table 4:** Summary of hydrodynamic diameter and PDI of samples measured on day 1 and day 5.

Sample	Time Evolution	Mean Hydrodynamic Diameter (nm)	PDI
L1	First Day	57	0.27
L2		98	0.24
L3		133	0.21
L4		174	0.19
L1	Fifth Day	66	0.26
L2		159	0.25
L3		238	0.23
L4		298	0.23

## Data Availability

The original contributions presented in the study are included in the article/Supplementary Materials. Further inquiries can be directed to the corresponding author/s.

## Supplementary Materials

The following supporting information can be downloaded at: https://www.mdpi.com/article/10.3390/nano16030212/s1, Figure S1: Fitting of the signal at 28.5°, corresponding to crystalline silicon, present in both the as-cast and irradiated colloids; Figure S2. Fitting of the signal at 31.8°, corresponding to crystalline SiO_2_ (cristobalite), present in both the as-cast and irradiated colloids; Figure S3. Fitting of the signal at 28.5°, corresponding to crystalline silicon present in both the as-cast and irradiated colloids after addition of silver salt; Figure S4. Fitting of the signal at 38.2° corresponding to the (111) crystalline direction of silver FCC. The Ag@SiO_2_ NPs were synthesized using both the as-cast and irradiated colloid; Figure S5: FTIR spectra of as-cast H_2_O-SiO_2_ colloid (red line), irradiated SiO_2_–H_2_O colloid (blue line) and Ag@SiO_2_ NPs using the irradiated colloid (magenta line); Figure S6. Band gap values obtained for all samples lie in the range of approximately 6.13–6.24 eV, which is consistent with the presence of predominantly SiO_2_ in the colloids. Table S1: Fitting results of SiO_2_-H_2_O colloid as cast and irradiated; Table S2: Results of the fittings of the signals corresponding to crystalline Si and Ag present in Ag@SiO_2_ NPs synthesized using both as-cast and irradiated SiO_2_–H_2_O colloids; Table S3: Williamson–Hall analysis parameters calculated for both as-cast and laser-irradiated SiO_2_–H_2_O colloids, as well as for the corresponding solutions after silver incorporation: microstrain, and dislocation density. Table S4. XRD vs. TEM Size Comparison [26].

## Abbreviations

The following abbreviations are used in this manuscript:

Ag@SiO_2_ NPs	Coated-silver nanoparticles
