# Supplementary material for: Study of the Optical, Structural and Electrophoretic Properties (Zeta Potential and Hydrodynamic Diameter) of SiO2-Coated Ag Nanoparticles
Source: Nanomaterials (Basel). 2026 Feb 6;16(3):212. doi: 10.3390/nano16030212 (PMC12899612; doi:10.3390/nano16030212)
Supplement: Supplementary file 1 [file nanomaterials-16-00212-s001.zip › nanomaterials-4072722-supplementary.pdf]

Article

Study of the optical, structural and electrophoretic properties (Zeta potential and Hydrodynamic Diameter) of SiO_2-coated Ag nanoparticles

Víctor E. Gámez-Albo[1], Ana B. López-Oyama[2,3,*], Eugenio Rodríguez González[1*], Jesús R. González-Castillo[4], Daniel Jímenez-Olarte[5], Deyanira Del Ángel-López[1], Elizabeth Reyna-Beltrán[6], Edgar G. Zamorano-Noriega[7]

1 Centro de Investigación en Ciencia Aplicada y Tecnología Avanzada-Unidad Altamira, Instituto Politécnico Nacional, Km 14.5 Carr. Puerto Industrial, Altamira 89600, Tamaulipas, Mexico; vgameza1900@alumno.ipn.mx (V.E.G.-A.); ddelangel@ipn.mx (D.D.Á.-L.)
2 Departamento de Investigación en Física (DIFUS), Universidad de Sonora, Blvd. Transversal S/N, Hermosillo 83000, Sonora, Mexico
3 Secihti-DIFUS, Universidad de Sonora, Blvd. Transversal S/N, Hermosillo 83000, Sonora, Mexico
4 Escuela Superior de Física y Matemáticas (ESFM—IPN), Instituto Politécnico Nacional, Mexico City 07738, Mexico; jrgonzalezc@ipn.mx
5 Escuela Superior de Ingeniería Mecánica y Eléctrica (ESIME—IPN), Instituto Politécnico Nacional, Mexico City 07738, Mexico; dajimenez@ipn.mx
6 Facultad de Medicina "Dr. Alberto Romo Caballero", Universidad Autónoma de Tamaulipas, Tampico 87000, Tamaulipas, Mexico; ereyna@docentes.uat.edu.mx
7 Departamento de Investigación en Polímeros y Materiales (DIPM), Universidad de Sonora, Blvd. Transversal S/N, Hermosillo 83000, Sonora, Mexico; a207217671@unison.mx
* Correspondence: ablopezoy@secihti.mx (A.B.L.-O.); eugenior62@gmail.com (E.R.G.)

X-Ray Diffraction supporting information for the article.

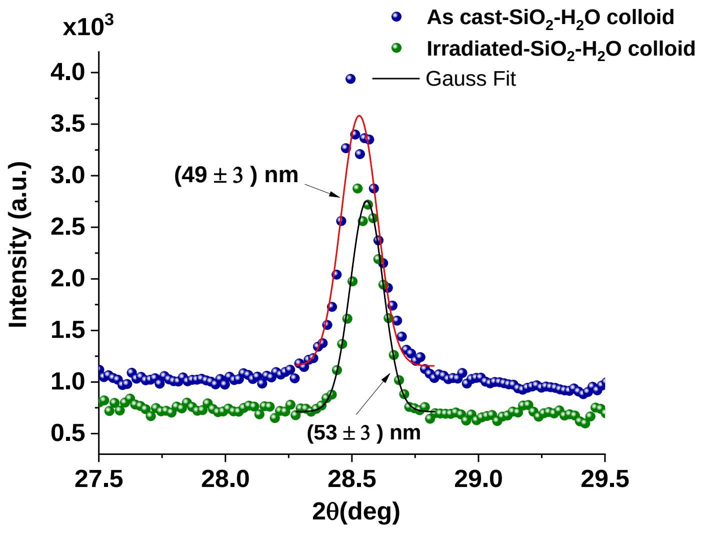

Figure S1. Fitting of the signal at 28.5° corresponding to crystalline silicon.

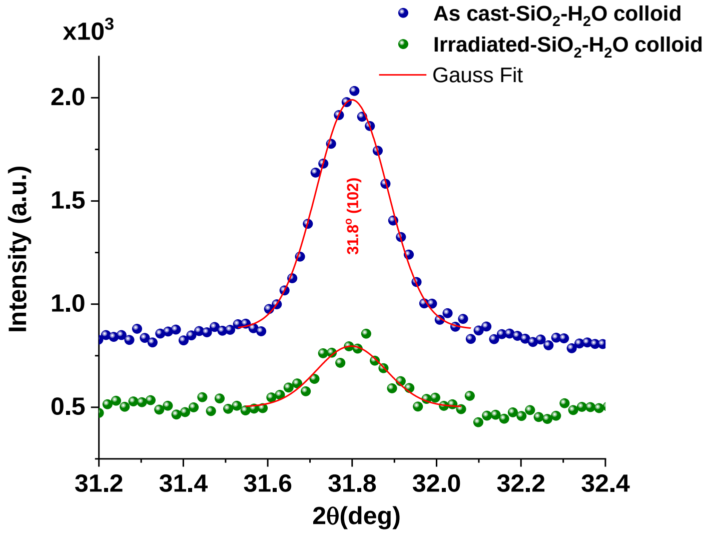

Figure S2. Fitting of the signal at 31.8° corresponding to the SiO_2 cristobalite.

Table S1. Fitting results of SiO_2-H_2O colloid as cast and irradiated.

Diffractogram	Material	2θ (°)	Peak área (a.u)	Peak Height	FWHM (°)	Dc (±3nm)
Si as cast	Crystalline Si	28.529	432	2425	0.16714	49
Si as cast	Crystalline SiO_2	31.7997	236	1110	0.1995	41
Si irrad	Crystalline Si	28.5587	331	2040	0.1525	53

Si irrad	Crystalline SiO_2	31.7982	61	294	0.1932	43

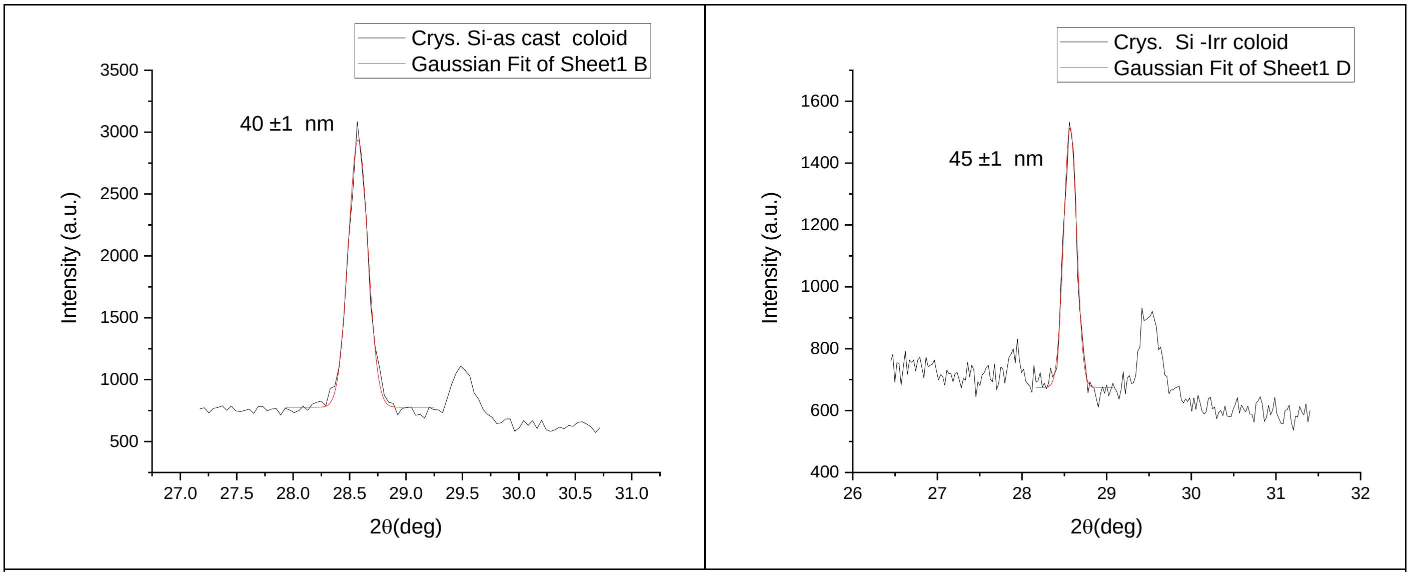

Figure S3. Fitting of the signal at 28.5° corresponding to crystalline silicon after addition of silver salt.

Deconvolution of the (111) silver signals located at 38.2° required two Gaussian components to differentiate the contribution of the smaller particles (<10 nm), which dominate the size distribution according to TEM analysis, from that of the larger ones (>10 nm).
Since the XRD peak intensity is proportional to the scattering volume—which in this case scales with the cube of the particle diameter, the scattering from a 20 nm particle is 64 times stronger than that from a 5 nm particle ($20^3/5^3 = 64$). Consequently, even a small fraction of larger particles in the size distribution can overshadow the contribution of the far more abundant smaller ones.

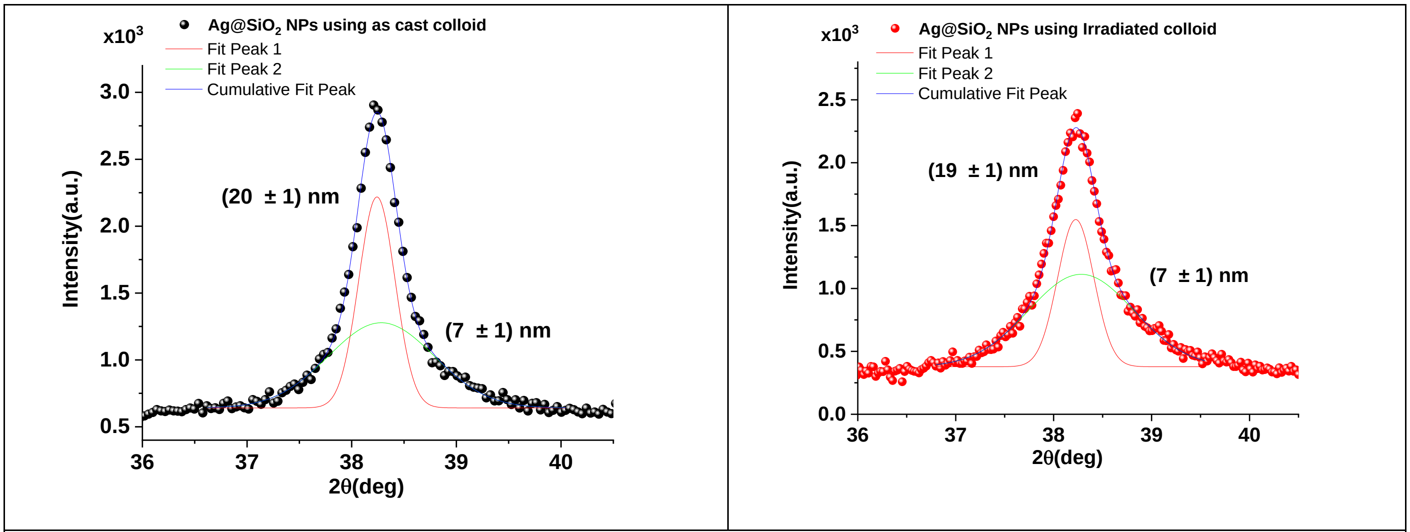

Figure S4. Fitting of the signal at 38.2° corresponding to the (111) crystalline direction of silver FCC using as cast and irradiated colloid.

Table S2. Fitting results of AgNPs using as cast and irradiated SiO_2.						
Diffractogram	Material	2θ (°)	Peak area (a.u)	Peak Height	FWHM (°)	Dc (±1nm)
$AgSiO_2$- using As cast colloid	Crystalline Si	28.5752	470		0.20348	40
	Ag1	38.2411	687	1576	0.4093	20
	Ag2	38.2829	810	636	1.1952	7
$AgSiO_2$- using irradiated colloid	Crystalline Si	28.568	159		0.17825	45
	Ag1	38.2260	543	1170	0.4357	19
	Ag2	38.2813	994	734	1.2715	7

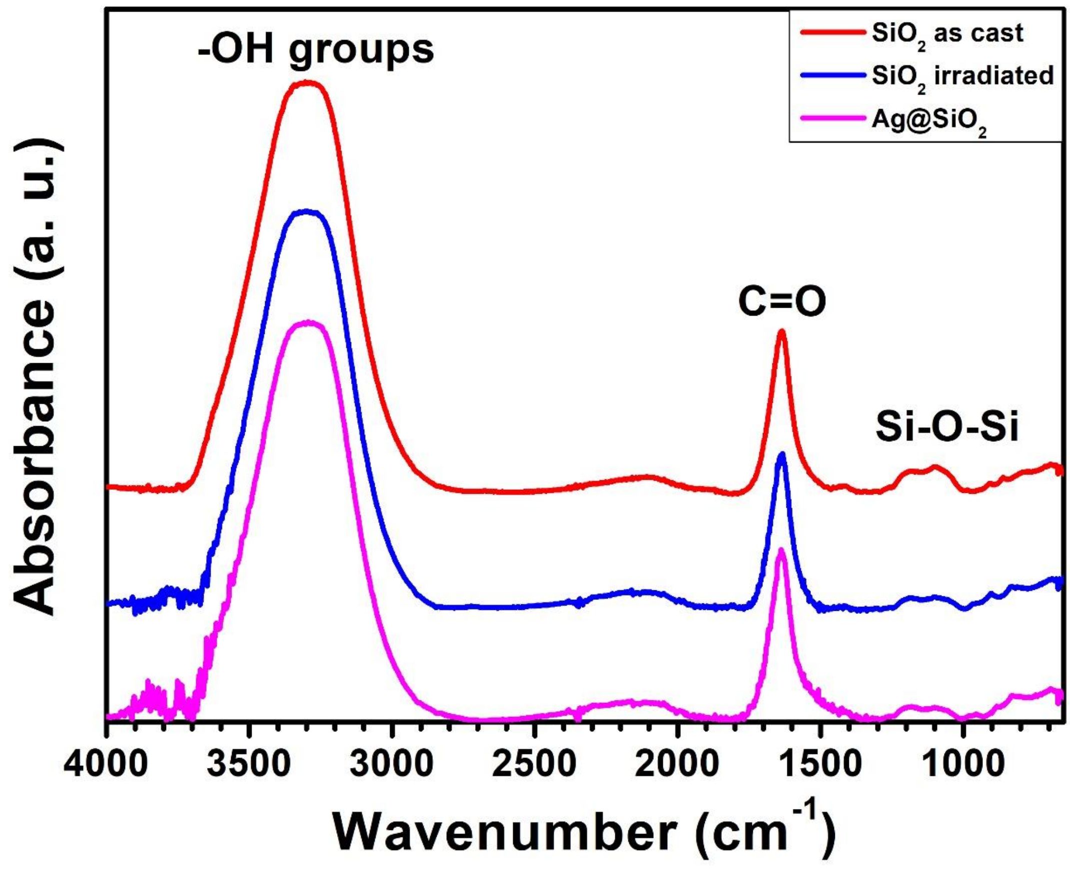

Figure S5: FTIR spectra of SiO_2 as cast (red line), irradiated SiO_2 (blue line) and Ag@SiO_2 (magenta line).

**Table S3. Williamson-Hall analysis parameters for pure SiO_2, irradiated SiO_2, and Ag@SiO_2 nanoparticles: microstrain, and dislocation density.**		
	**Microstrain (ε) 1 x 10^{-3}**	**Dislocation density 1 x 10^{-2}**
Ag@SiO_2 as cast	1.28	0.57
Ag@SiO_2 irrad	2.72	0.30
SiO_2 as cast	0.02	0.25
SiO_2 irrad	0.59	0.36

of Ermakov et.al. [1]

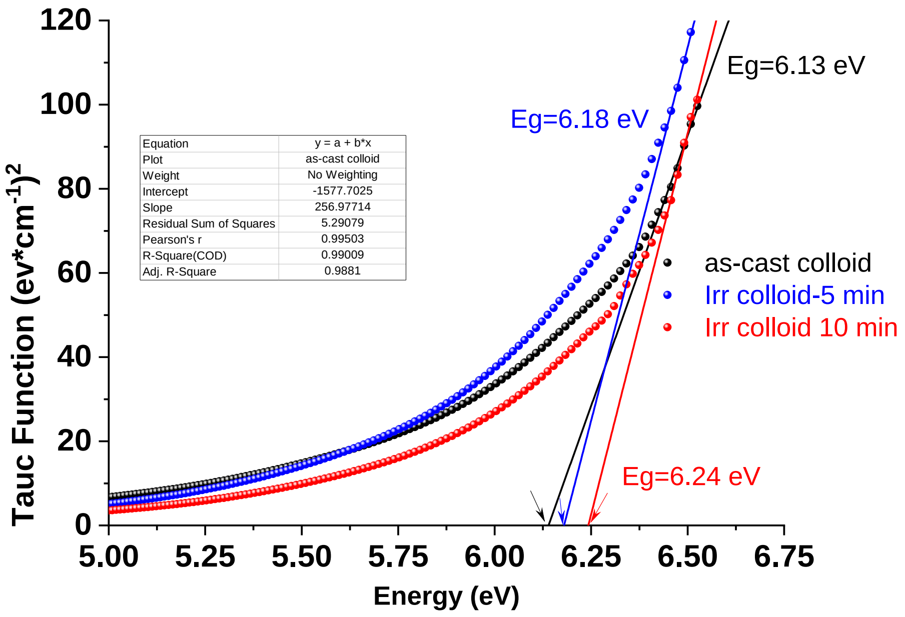

Figure S6. Band gap values obtained for all samples lie in the range of approximately 6.13–6.24 eV, which is consistent with the presence of predominantly SiO_2 in the colloids.

Table S4. XRD vs TEM Size Comparison

Technique	Ag Core Size	Method
XRD	20 ± 1 nm (larger particles distribution)<br>7 ± 1 nm (smaller particles distribution)	Scherrer eq. (111) peak
TEM	15.4 ± 5.2 nm	> 500 particle statistics
